# Knowledge, attitudes, and practices of healthcare workers towards COVID-19 patients in the United Arab Emirates: a cross-sectional study

**DOI:** 10.1186/s12913-023-09215-y

**Published:** 2023-03-14

**Authors:** Amina M. Al Marzouqi, Michael Ekubu Otim, Leena S. Kehail, Ramsha A. Kamal

**Affiliations:** 1 College of Health Sciences, University of Sharjah, Sharjah, United Arab Emirates; 2 Department of Public Health and Behavioral Sciences, Dubai Medical College for Girls, Dubai, United Arab Emirates

**Keywords:** COVID-19 knowledge, COVID-19 attitudes, COVID-19 Practices, COVID-19, Healthcare workers, United Arab Emirates

## Abstract

**Background:**

The Coronavirus disease (a highly infectious viral disease) quickly swept across the globe in 2020, causing mortality and severe respiratory illnesses. It quickly affected businesses and publicly provided services in United Arab Emirates (UAE), imposing significant costs to society. The general population of UAE was jittery and unsure how to address the problem. The focus turned on government and Health Care Workers (HCW) to handle the pandemic. Thus, knowledge, attitudes and practices (KAP) of HCW became critical for the treatment and isolation of COVID-19 patients. Thus, the KAP of the HCW came under scrutiny. This is study set out to answer the research question, by investigating the KAP of HCW related to COVID-19 in the UAE.

**Methods:**

This was a cross-sectional study undertaken in UAE. The focus of was on HCWs as a population. Using convenience sampling with the help of Creative Research Software, the participants were identified, and an online questionnaire (Google Forms) distributed by the author. The questionnaire was adapted from the instrument developed by Bruce and Frey. It contained questions on demographic variables, knowledge, attitudes, and practice of HCW related to COVID-19. That instrument was contextualized to the UAE society and validated.

**Results:**

Among the participants in the study, the majority, 90.7% (97) knew that the absence of fever did not mean there was no chance of transmission from an infected person. Further, 84.1% (90) agreed that wearing general medical face masks helps prevent one from contracting COVID-19. However, only 36.4% (39) strongly believed that wearing a well-fitting face mask was effective. In addition, only 15.9% (17) reported confidently managing patients with symptoms of COVID-19, while 54.2% (58) indicated they were afraid of contracting the virus from patients. Almost 50% of the participants noted that they avoided patients who had symptoms of the COVID-19.

**Conclusion:**

This study revealed that the KAP for healthcare workers from UAE healthcare facilities related to COVID-19 was high. The healthcare workers were trained well and that positively affected awareness and the practice of HCW regarding the spread of the virus.

## Background

The ongoing spreading of COVID-19 and the emergence of variants of the highly contagious respiratory disease (COVID-19) has threatened global health [1]. The presence of pneumonia led to the identification of the COVID-19, and close monitoring of the virus suggested a mean incubation period of 5 days [2]. The novel coronavirus is structurally related to the virus that causes severe acute respiratory syndrome (SARS) and hence the name of the disease, SARS-Cov-19 (COVID-19). Several variants have emerged since this data was collected in February 2021. These include Delta, Omicron and others [3].

COVID-19 spreads via the respiratory secretions of infected persons when they cough or sneeze, and it is a highly infectious disease. In early 2020, several countries were jittery and unsure of what to do about COVID-19, resulting in different policy interventions which were knee-jerk reactions. Supportive healthcare interventions (including now vaccines) are available to hospitalized patients. Social distancing, wearing a face mask, washing hands, or using alcohol-based sanitizers, and staying home when experiencing any symptoms can prevent the spread of the virus. Older people and those with chronic disease conditions such as diabetes or respiratory infections are more likely to develop severe symptoms and higher mortality [4]. The global mortality rate is about 2% [5]. In UAE, COVID-19 has negatively affected many sectors of the economy, including health, business, education, aviation, tourism, and others. In Africa, a study in Eastern Democratic Republic of Congo reported that COVID-19 had adversely affected the Congo economy more than the Ebola disease despite the lower mortality rate of COVID-19 [6]. HCW were adversely affected, with staff morale affected by salary cuts and employment terminations [7].

Preventive measures such as social distancing and lockdowns have resulted in higher costs of living. Transporting goods for example has been catastrophic for emerging economies such as Nigeria [8]. During the second quarter of 2020, commercial travel declined by 75% and shipping by almost 50% on average from 75% in mid-April 2020 [9, 10] The education sector also suffered adverse consequences from the Pandemic, including temporary school closures, which affected about 60% of students globally [11]. The UAE Al Qassimi Foundation indicated that students, staff, and parents experienced high stress during this time [12]. Working parents and students with special needs received free internet packages and tutorials [13].

The Impact of the COVID-19 on the healthcare sector has affected social relationships through fear and anxiety about being separated, infected and the risk of losing family members [14]. This disease has had significant impact on capacities of hospitals and other healthcare facilities to do their jobs in general in USA, just like in the UAE [15, 16]. In response, hospitals have adopted better hygiene practices, and practices to reduce their health workers from COVID-19 infection.

A study by Shaukat and colleagues noted that healthcare workers handling COVID-19 cases are at an increased risk of negative impacts on their physical and mental health [17]. Their scoping review found that the COVID-19 infection was linked to multiple factors, including working in high-risk areas and closely contacting patients (for example, over 12 times per day for more than 15 h). Furthermore, they found common symptoms among healthcare workers include fever (85%), cough (70%), fatigue (70%), and skin damage (97%). Healthcare workers experienced high levels of depression, anxiety, insomnia, and distress, with nurses and female healthcare workers being highly affected. Given those findings above, the authors became interested in investigating how the UAE HCW were reacting/coping with the Covid-19 Pandemic.

### Aim

The primary aim of this study was to answer the following question: identify the KAP of healthcare professionals handling the COVID-19 patients in the UAE?

### Objectives

1. What is the knowledge of HCW about COVID-19?
2. What are the attitudes of HCW towards COVID-19 patients or suspected patients.
3. What is the KAP of HCW towards COVID-19 patients?

## Methods and materials

### Study Design and setting

A cross-sectional study design was used to undertake this study in February 2021. The participants were from Sharjah, Dubai, Abu Dhabi, Ajman, and Umm al Quwain health facilities. The authors did not receive responses from Ras Al Khaimah and Fujairah due to challenges in communication lock-down measures to control the spread of disease. The respondents were from different specialties, such as physicians, radiographers, nurses, and pharmacists.

#### Inclusion criteria

All HCW who are trained as such were invited to undertake the study. All HCW who work with COVID-19 or suspected COVID patients were included for the study.

#### Exclusion criteria

Those who were not directly involved with the patients were not included. These included the management and technicians, and others.

### Population and sample size

The target population comprised healthcare workers in the UAE who were likely to encounter patients with confirmed or suspected COVID-19 in 2021. Using the 2017 estimate (113,000), of the number of healthcare workers in the UAE to estimate the number of healthcare workers in 2020 [18]. However, not all the HCW face potential COVID-19 patients. A software called Creative Research Systems was used to estimate the sample size and came up with a size of 384 [19]. However, due to these challenges related to the Pandemic, we received responses from only 107 respondents. Moreover, majority of the participants were from Dubai.

### Data collection

Using an online survey developed by Bruce [20], we contextualized the survey to the UAE setting. A group of students and faculty were surveyed to assess the appropriateness of the question was done. Participants completed the questionnaires online using Google Forms, and they were asked to forward to the people they knew and had their email addresses.

Convenience sampling technique was used to recruit the participants for this study [20]. This technique allowed the generation of a pool of participants through referrals with particular characteristics as a target population. The technique is a non-probability sampling technique use by Bruce, et al.; Rao, et al. Rabanni and Saul; and in which the identified study participants recruit future participants from among their connections [20–22].Due to the COVID-19 restrictions and related issues, healthcare workers were asked to complete the survey and forward the link to their colleagues in other healthcare facilities [23].

### Data analysis

We used Statistical Package for Social Sciences (SPSS) version 21 to analyze the data [24]. Results were presented as descriptive statistics (namely, means, frequencies, and percentages). We also used cross-tabulation to display the relationships between the participants’ demographic variables and responses to the KAP variables. The independent t-test and ANOVA were not done because the data was not continuous, and the sample was small. This creates a limitation in this study.

### Ethical considerations

This study followed the procedures set out in the Declaration of Helsinki. Ethical approval was obtained from the University of Sharjah Research Ethics Committee (ID: REC-21-02-21-02-S). We sent information about the survey to the participants. We advised them that clicking on the survey link meant that they had consented to participate.

## Results

### Demographic characteristics

Of all the respondents, approximately 92% of the participants worked in public healthcare facilities. The mean age of the participants was 40.5 ± 11 years, and 67.3% were female. Most of the participants (87%) worked in the Emirate of Dubai. The participants were classified by occupation as follows: specialist physicians (16.8%), radiographers (43%), nurses (23.4%), pharmacists (8.4%), and other (8.4%). Most of them had a bachelor’s degree (66.4%) as their highest level of education (Table 1).

**Table 1:** Demographic characteristics of the participants

Characteristic	Variable	n (%)
**Workplace**	Private	9 (8.4)
	Public	98 (91.6)
**Emirate**	Dubai	93 (86.9)
	Sharjah	9 (8.4)
	Others	5 (4.6)
**Gender**	Male	35 (32.7)
	Female	72 (67.3)
**Age, years**	21–30	21 (19.6)
	31–40	37 (34.6)
	41–50	24 (22.4)
	51–60	25 (23.4)
**Qualification**	Specialist physician	18 (16.8)
	Radiographer	46 (43.0)
	Nurse	25 (23.4)
	Pharmacist	9 (8.4)
	Other	9 (8.4)
**Education**	Diploma	139 (12.1)
	Bachelor’s degree	71 (66.4)
	Master’s degree	16 (15.0)
	Doctor of Philosophy	7 (6.5)
**Nationality**	Philippines	30 (28.0)
	India	30 (28.0)
	UAE	14 (13.1)
	Other	33 (30.8)
**Source of information on COVID-19 (more than one source may be identified)**	International health organizations sites such as the WHO and CDC	84 (78.5)
	Government sites (such as Ministry of Health)	91 (85)
	News media (such as Televisions and radio)	57 (53.3)
	Social media (such as WhatsApp and Facebook)	51 (47.7)
	Journals	32 (29.9)

### Knowledge

The participants’ responses regarding their knowledge of the COVID-19 are presented in Table 2. Most of the participants (94%) were aware that there was no cure for the COVID-19, but that early diagnosis and supportive interventions would help patients recover faster. Most of the participants (78.5%) disagreed with the statement that eating contaminated food could cause Covid-19. In addition, 91% agreed that the absence of a fever does not mean that individuals with the COVID-19 could not infect others. In terms of virus spread, 94.4% of the participants knew that the COVID-19 spreads through the respiratory droplets of infected individuals, and 84.1% agreed with the statement that wearing a general medical mask helps prevent one from contracting COVID-19. Most of the participants (93%) agreed that children and young adults need to take preventive measures against the COVID-19, and 90% indicated that individuals should avoid crowded places to reduce the possibility of COVID-19 infection. 96% of the healthcare workers knew that the isolation of infected patients for 14 days is an effective way to reduce the spread of the virus.

**Table 2:** Knowledge about COVID-19 among healthcare workers in the United Arab Emirates

Variable	n (%)
Major clinical symptoms of COVID-19	
Fever	107 (100)
Headache	91 (85)
Myalgia (muscle pain)	80 (74.8)
Smell disturbance	87 (81.3)
Sore throat	98 (91.6)
Diarrhea	71 (66.4)
Runny nose	61 (57)
Sneezing	56 (52.3)
Cough	104 (97.2)
Confusion	23 (21.5)
There is currently no cure for COVID-19.	
True	100 (93.5)
False	4 (3.7)
I don’t know	3 (2.8)
Not all COVID-19 patients will develop severe illness, only those with chronic illnesses.	
True	84 (78.5)
False	19 (17.8)
I don’t know	4 (3.7)
Contaminated food and seafood could result in COVID-19 infection.	
True	23 (21.5)
False	68 (63.6)
I don’t know	16 (15.0)
COVID-19 patients cannot transmit the virus when a fever is not present.	
True	5 (4.7)
False	97 (90.7)
I don’t know	5 (4.7)
The COVID-19 virus spreads via the respiratory droplets of infected individuals.	
True	101 (94.4)
False	3 (2.8)
I don’t know	3 (2.8)
Wearing a mask can help prevent one from acquiring COVID-19 virus.	
True	90 (84.1)
False	14 (13.1)
I don’t know	3 (2.8)
Children and young adults should avoid becoming infected with COVID-19.	
True	9 (8.4)
False	94 (87.9)
I don’t know	4 (3.7)
Individuals should avoid going to crowded places to prevent COVID-19 transmission and infection.	
True	96 (89.7)
False	10 (9.3)
I don’t know	1 (0.9)
People who are infected with COVID-19 should be isolated and treated.	
True	103 (96.3)
False	3 (2.8)
I don’t know	1 (0.9)
Contact with someone with COVID-19 requires 14 days of observation.	
True	102 (95.3)
False	3 (2.8)
I don’t know	2 (1.9)

### Attitudes

Table 3 presents the participants’ responses regarding their attitudes towards COVID-19 patients. Approximately 25% agreed that the younger population groups do not contract the COVID-19. Most of the participants (90%) agreed or strongly agreed that wearing a well-fitting mask is effective in helping prevent individuals from contracting the virus. 88% agreed or strongly agreed that washing hands with soap could help prevent one from being infected with the virus (Table 3). Only 44% of the participants agreed that they confidently participated in managing patients who had signs and symptoms of COVID-19, with 50.5% afraid of carrying the virus from their workplace to home and 54.2% fearful of contracting the virus from patients. However, most participants had confidence in their workplaces to be clean from the COVID-19. Interestingly, 55.1% agreed, and 29.9% strongly agreed that the UAE was in position to contain the virus.

**Table 3:** Attitudes toward COVID-19 among healthcare workers in the United Arab Emirates

Variables	n (%)
Young population groups are protected against COVID-19	
Strongly disagree	32 (29.9)
Disagree	29 (27.1)
Not sure	19 (17.8)
Agree	17 (15.9)
Strongly agree	10 (9.3)
Wearing a well-fitting face mask is effective in helping prevent COVID-19.	
Strongly disagree	6 (5.6)
Disagree	2 (1.9)
Not sure	3 (2.8)
Agree	57 (53.3)
Strongly agree	39 (36.4)
Washing with soap can help prevent you from becoming infected with COVID-19.	
Strongly disagree.	5 (4.7)
Disagree	4 (3.7)
Not sure	4 (3.7)
Agree	60 (56.1)
Strongly agree	34 (31.8)
When a patient has signs/symptoms of COVID-19, I can confidently participate in his management	
Strongly disagree	10 (9.3)
Disagree	15 (14.0)
Not sure	18 (16.8)
Agree	47 (43.9)
Strongly agree	17 (15.9)
I am afraid of carrying COVID-19 virus from my workplace to my home.	
Strongly disagree	3 (2.8)
Disagree	7 (6.5)
Not sure	11 (10.3)
Agree	54 (50.5)
Strongly agree	32 (29.9)
I am afraid of contracting COVID-19 virus from patients.	
Strongly disagree	4 (3.7)
Disagree	18 (16.8)
Not sure	8 (7.5)
Agree	58 (54.2)
Strongly agree	19 (17.8)
My workplace is adequately equipped to curb the spread of COVID-19 Virus.	
Strongly disagree	2 (1.9)
Disagree	9 (8.4)
Not sure	16 (15.0)
Agree	53 (49.5)
Strongly agree	27 (25.2)
My workplace is adequately equipped to protect its employees from COVID-19 virus .	
Strongly disagree	3 (2.8)
Disagree	11 (10.3)
Not sure	8 (7.5)
Agree	59 (55.1)
Strongly agree	26 (24.3)
The UAE is in an appropriate position to contain COVID-19.	
Strongly disagree	2 (1.9)
Disagree	1 (0.9)
Not sure	13 (12.1)
Agree	59 (55.1)
Strongly agree	32 (29.9)

### Practices

The healthcare workers’ practices related to the COVID-19 among in the UAE requires a better understanding of the factors influencing the spread of the COVID-19 and attitudes of the workers. The participants’ responses concerning COVID-19 practices are presented in Table 4. Almost all the participants (n = 104; 97.2%) always wore masks during patient contact, 85% (n = 91) always refrained from shaking hands, and 91.6% n= (98) always washed their hands before and after handling each patient. Most of the participants (n = 92; 86.0%) indicated that they always educated their patients about COVID-19 preventive measures, and 95.3% (n = 102) always obeyed all government rules related to COVID-19. 94.4% of the participants indicated that they always maintained social distancing. Furthermore, 26.2% (n = 28) of the respondents occasionally tried to avoid patients with COVID-19 symptoms, while 24.3% (n = 26) never tried to prevent such patients. Surprisingly, 49.5% (n = 53) of the participants indicated that they always avoided patients with signs and symptoms of COVID-19, given how professional healthcare workers in UAE are.

**Table 4:** COVID-19 practices among healthcare workers in the UAE

Practices	N (%)
I wear a mask when in contact with patients.	
Always	104 (97.2)
Occasionally	1 (0.9)
Never	2 (1.9)
I refrain from shaking hands with people.	
Always	91 (85.0)
Occasionally	3 (2.8)
Never	13 (12.1)
I wash my hands before and after handling each patient.	
Always	98 (91.6)
Occasionally	8 (7.5)
I always maintain social distancing.	
Always	101 (94.4)
Never	6 (5.6)
I avoid patients with signs and symptoms suggestive of COVID-19	
Always	53 (49.5)
Occasionally	28 (26.2)
Never	26 (24.3)
I obey all the government’s rules related to COVID-19.	
Always	102 (95.3)
Occasionally	5 (4.7)
Never	0 (0.0)
I educate my patients about COVID-19 preventive measures.	
Always	92 (86.0)
Occasionally	13 (12.1)
Never	2 (1.9)

Table 5 shows the KAP and the participants’ age groups. We found that the majority of participants 30% (n = 39) in the 31–40-year age group believed that a person without a fever could not transmit COVID-19 to others was genuine statement. N = 92 (86%) healthcare workers always educate their patients about COVID-19 preventive measures. Unfortunately, n = 81 (75.70%) workers confessed to having always or occasionally avoided patients with Covid-19 signs and symptoms.

**Table 5:** Association between COVID-19 knowledge, attitudes, and practices and age among healthcare workers in the UAE

Variables		Age, years				Total
		21–30	31–40	41–50	51–60	
People with COVID-19 cannot transmit the virus to others when a fever is not present.	True	0	5	0	0	5
	False	21	29	22	25	97
	I don’t know	0	3	2	0	5
Total		21	37	24	25	107
When a patient has signs and symptoms of COVID-19, I can confidently participate in their management.	Strongly Disagree	2	5	1	2	10
	Disagree	2	4	6	3	15
	Not Sure	3	7	6	2	18
	Agree	9	15	10	13	47
	Strongly Agree	5	6	1	5	17
Total		21	37	24	25	107
In recent days, I have avoided patients with Covid-19 signs and symptoms.	Always	10	22	11	10	53
	Occasional	9	10	5	4	28
	Never	2	5	8	11	26
Total		21	37	24	25	107

## Discussion

This study highlighted the KAP of healthcare professionals when handling the COVID-19 patients in the UAE. This was necessary, given that, most HCW in the UAE are migrant workers with different healthcare worker levels and training. These HCW have different attitudes due to different beliefs and cultural orientations, which affects their attitudes towards COVID-19. And finally how the healthcare workers practiced healthcare when attending to COVID-19 patients or those suspected of having clinical signs of COVID-19, reflects their attitudes and knowledge.

This study showed that the healthcare workers in the UAE had very good knowledge about COVID-19 and related viruses. For example, most were aware that although COVID-19 has no cure, early diagnosis and supportive care can help most patients recover [25]. Furthermore, most participants understood that the absence of fever did not the lack of COVID-19. This knowledge could be due to the consistent ongoing provision of continuous education and support for healthcare professionals to improve their knowledge on different diseases and infectious diseases in particular. This will help strengthen the UAE’s response to the current Pandemic and similar public health issues that had risen or likely to arise in the future. In our study, only a few participants 8.4% believed that children and young adults could avoid infection with the COVID-19, consistent with the findings of Ferdous et al., who reported that 4% of the respondents in their study held this belief [26]. Encouragingly, as for the Olum et al. study [25], most of the participants in our research agreed/strongly agreed that wearing a well-fitting mask is effective in helping prevent COVID-19 infection. The level of knowledge of the HCW and frequent reminders via official international and governmental websites may explain the relatively high rate of knowledge about the protective effect of masks. To enforce the use of masks shows, the UAE vigilance and enforcement, resulting to penalties for individuals who were non-compliant with this requirement.

Healthcare workers can play a role in guiding the public about practicing precautionary measures to help reduce the spread of the disease and demystifying the COVID-19 [26]. In our study, 80% of the participants believed that avoiding crowded places helps protect people from COVID-19 infection, 9.3% did not share this view. Strong adherence to social distancing and wearing face masks, considered effective in combatting COVID-19 spread, were followed by the HCW. Healthcare workers in the older age groups were particularly conscious of the risk of COVID-19 transmission. This is consistent with scientific advice that older people are susceptible to severe COVID-19 outcomes as many tend to have preexisting conditions.

Overall, the healthcare workers in our study adhered to the government’s rules and guidelines regarding preventive measures (Table 4). In our study, most of the healthcare workers’ attitudes towards their patients about COVID-19 preventive measures were very positive. In fact, 60% (n = 64) of the healthcare workers were confident to participate in the management of their patients even when patients had signs and symptoms of COVID-19. For example, 92% of the participants in Ferdous et al.’s study were afraid of carrying the virus home. The UAE HCW established the National Crisis and Emergency Management Authority [27], to establish field hospitals and devise comprehensive plans to respond to the COVID-19, including free PCR testing and, later, free vaccinations for the population. WHO and related institutions have guided countries on their responses to COVID-19 during the pandemic, worked hard to change attitudes of healthcare workers and those of the general publics. For example, the impact of COVID-19 on Mental and physical health could be alleviated using training materials for health care workers in many countries and provided announcements and guidelines to encourage people to adhere to public health measures [28].

However, the key limitation of our study is that the findings cannot be generalized due to the small sample size - not being representative. We argue that there is still a lot to learn since the next key task is to ensure that every resident should receive a vaccine without hesitancy. Another limitation is that majority of the participants were from Dubai. This is mainly because of social restrictions employed by government to curb the spread of the virus. Other restrictions at the time of the study hindered data collection, potentially excluding survey participants who did not have internet access. Furthermore, some confounders were not identified and measured. The UAE government was very vigilant at monitoring and controlling the spread of the virus, hence the very good KAP from the HCW.

## Conclusion

COVID-19 brought about unprecedented economic and social challenges globally. This led to UAE government to strengthen its efforts at enforcing public health interventions to curb the spread and infection of the virus through restrictions and precautionary guidelines. Coupled with high KAP among healthcare workers, the HCW performed very well. The healthcare workers in this study believed that mask and social distancing protocols could help prevent them from becoming infected with the COVID-19. Could this due to government vigilance or they found out themselves. However, it was not easy at that point to attribute the proportion of HCW willingness to use face masks and maintain social distancing to the knowledge or attitudes of healthcare workers beyond government regulations.

## Data Availability

The datasets generated during the present study are not publicly available because of ethical restrictions but are available from the corresponding author on reasonable request.
